# Effects of the lipid regulating drug clofibric acid on PPARα-regulated gene transcript levels in common carp (*Cyprinus carpio*) at pharmacological and environmental exposure levels

**DOI:** 10.1016/j.aquatox.2015.01.033

**Published:** 2015-04

**Authors:** Jenna Corcoran, Matthew J. Winter, Anke Lange, Rob Cumming, Stewart F. Owen, Charles R. Tyler

**Affiliations:** a University of Exeter, Biosciences, College of Life & Environmental Sciences, Exeter EX4 4QD, UK; b AstraZeneca Global Environment, Brixham Laboratory, Freshwater Quarry, Brixham TQ5 8BA, UK

**Keywords:** Peroxisome proliferator activated receptor α (PPARα), Acyl-coA oxidase (Acox1), Clofibric acid, Fibrate, Lipid metabolism, Teleost fish, Peroxisomal β-oxidation, Bile acid synthesis, Drug metabolism

## Abstract

In mammals, the peroxisome proliferator-activated receptor α (PPARα) plays a key role in regulating various genes involved in lipid metabolism, bile acid synthesis and cholesterol homeostasis, and is activated by a diverse group of compounds collectively termed peroxisome proliferators (PPs). Specific PPs have been detected in the aquatic environment; however little is known on their pharmacological activity in fish. We investigated the bioavailability and persistence of the human PPARα ligand clofibric acid (CFA) in carp, together with various relevant endpoints, at a concentration similar to therapeutic levels in humans (20 mg/L) and for an environmentally relevant concentration (4 μg/L). Exposure to pharmacologically-relevant concentrations of CFA resulted in increased transcript levels of a number of known PPARα target genes together with increased acyl-coA oxidase (Acox1) activity, supporting stimulation of lipid metabolism pathways in carp which are known to be similarly activated in mammals. Although Cu,Zn-superoxide dismutase (Sod1) activity was not affected, mRNA levels of several biotransformation genes were also increased, paralleling previous reports in mammals and indicating a potential role in hepatic detoxification for PPARα in carp. Importantly, transcription of some of these genes (and Acox1 activity) were affected at exposure concentrations comparable with those reported in effluent discharges. Collectively, these data suggest that CFA is pharmacologically active in carp and has the potential to invoke PPARα-related responses in fish exposed in the environment, particularly considering that CFA may represent just one of a number of PPAR-active compounds present to which wild fish may be exposed.

## Highlights

• CFA appears to have a low propensity to bioconcentrate and has a plasma half-life of <4 days in carp.
• CFA increases levels of mRNA of a number of genes known to be regulated by PPARα in mammals.
• PPARα activation changes levels of mRNA of genes involved with several detoxification/ biotransformation system components in carp.
• CFA alters levels of mRNA and activity of the inducible β-oxidation pathway enzyme Acox1, a known indicator of peroxisome proliferator exposure.

## 1 Introduction

Peroxisome proliferator-activated receptors (PPARs) are transcription factors activated endogenously by fatty acids and their derivatives and are well studied in mammals due to their potential as therapeutic targets for dyslipidaemia, obesity and diabetes.

There are three known PPAR subtypes, α, -β and -γ, which have different roles in lipid and energy dynamics, show differential expression patterns and they have different downstream gene targets. PPARα plays a role in clearance of circulating lipids via regulation of the expression of genes involved in lipid metabolism, PPARβ is involved in lipid oxidation and cell proliferation, and PPARγ promotes adipocyte differentiation to enhance blood glucose uptake (Singh et al., 2011).

In mammals, PPARα mediates the action of a structurally diverse group of compounds known as peroxisome proliferators (PPs). PPs bind to the PPARα which in turn binds response elements (PPREs) in the promoter region of target genes that include various enzymes involved in lipid metabolism (Staels et al., 1998). Of these, the most notable is acyl-coA oxidase (Acox1), which has an important role in peroxisomal *β* oxidation. Others include enzymes involved in other stages of fatty acid catabolism via both peroxisomal and mitochondrial β-oxidation pathways (including several cytochrome P450 (CYP) 4 isoforms), lipoprotein metabolism, triglyceride clearance (*e.g.*, lipoprotein lipase) and cholesterol catabolism (CYP7A1 and CYP27A). PPREs have also been identified in the promoter regions of a number of genes involved in inflammation, gluconeogenesis, biotransformation, ketogenesis, lipogenesis, and amino acid metabolism (Mandard et al., 2004). In some species, PPs cause peroxisome proliferation and have been associated with oxidative stress and hepatocarcinogenesis resulting from an increased production of hydrogen peroxide (H_2_O_2_) (Rao and Reddy, 1991). PPARα is targeted therapeutically in humans by fibrate drugs, which are used clinically to lower blood plasma lipid levels, via transcriptional activation of a number of genes regulated by PPARα (Staels et al., 1998). As with many of the orphan nuclear receptors, PPAR has been well conserved during vertebrate evolution. Fish have three isoforms analogous to mammalian PPARα, -β and -γ (Andersen et al., 2000; Maglich et al., 2003; Boukouvala et al., 2004; Leaver et al., 2005) which in some species are present as multiple isoforms of individual subtypes (Leaver et al., 2005) due to evolutionary gene duplication events (Urbatzka et al., 2013). In common with rodents, high levels of expression of PPARα has been reported in the liver of several fish species (Ibabe et al., 2002; Ibabe et al., 2004; Leaver et al., 2007; He et al., 2012) probably due to the organ’s prominent role in fatty acid oxidation (Colliar et al., 2011).

A wide range of chemicals discharged into the environment are known to interact with PPARα, including certain pharmaceuticals (*e.g.*, the fibrates), phthalate ester plasticizers, PAHs, PCBs, alkylphenols, and some pesticides (Gibson, 1993). Complex effluent discharges from bleached kraft pulp and paper mills have also been shown to induce peroxisome proliferation in fish and other aquatic species (Cajaraville et al., 2003). Despite this, there are relatively few published data on the activity of PPs in fish, especially with respect to downstream effects on genes important in hepatic metabolism and detoxification.

*In vitro* reporter gene assays have previously shown that hypolipidemic drugs are agonists of teleost (rainbow trout, in particular) PPARs (Liu et al., 2005) and consequently, we investigated responses in the PPARα system in carp (*Cyprinus carpio*) exposed, *in vivo*, to clofibric acid (2-(4)-chlorophenoxy-2-methyl propionic acid; CFA) the active metabolite of the lipid lowering fibrate clofibrate. First marketed in the US in 1967, clofibrate was widely prescribed in humans and is still commonly detected in the aquatic environment at concentrations ranging from high ng/L to low μg/L in sewage effluents and surface waters (Stan and Linkerhagner, 1992; Stan et al., 1994; Heberer et al., 1995; Buser et al., 1998; Ternes, 1998; Tixier et al., 2003; Zorita et al., 2008). CFA has also been detected in groundwater and drinking water at levels up to 4 μg/L and 270 ng/L, respectively, (Heberer and Stan, 1997) illustrating that it is highly mobile and persists in the environment. Indeed, CFA is regarded as a relatively persistent drug residue with an estimated lifespan in the environment of 21 years (Buser et al., 1998; Zuccato et al., 2000; Winkler et al., 2001; Tixier et al., 2003).

In this study, two exposure concentrations of CFA were selected for studies into the effects of this drug in fish, specifically the carp, *C. carpio*. The first concentration (20 mg/L in the exposure water) was chosen in an attempt to generate a pharmacologically-relevant concentration in the exposed fish which based on previous data and reported human therapeutic plasma concentrations is between 50 and 250 μg/mL (Regenthal et al., 1999). This concentration was chosen to examine the potential for mode of action-related effects, in a non-target, environmentally-relevant species. The second concentration employed (4 μg/L) was selected to establish whether such effects occurring for therapeutic doses could occur at an exposure level comparable with measured concentrations of CFA in the aquatic environment. For each treatment three sampling points were employed, each incorporating the measurement of plasma CFA levels to assess bioavailability and propensity for persistence in carp.

Transcriptional responses to CFA exposure in carp were assessed via temporal changes in mRNA levels of hepatic *ppara* and target genes known to be regulated via PPARα in mammals. These included genes encoding acyl-coA oxidase (*acox1),* thiolase (*acaa1)* and cytochrome P450 4 (*cyp4)* which are involved in lipid metabolism; sterol 27-hydroxylase (*cyp27a)* and apolipoprotein-A1 (*apoa1)* which play a role in cholesterol homeostasis; lipoprotein lipase (*lpl*) involved in lipoprotein metabolism; and Cu/Zn superoxide dismutase (*sod1)* a cellular antioxidant. Acyl-coA oxidase (Acox1) activity, and Cu,Zn superoxide dismutase (Sod1), a key antioxidant enzyme controlled via the PPARα in mammals, were also measured. The *in vivo* mRNA levels of several key genes encoding proteins involved in hepatic drug metabolism (*cyp2k*, *cyp3a*, *gsta*, *gstp*) and transport (*mdr1, mrp2*) were also measured as we have shown previously that these are responsive to CFA in cultured carp hepatocytes. The liver was the main target tissue for studies on gene responses in the PPARα pathways due to its role in fatty acid metabolism.

## 2 Materials and methods

### 2.1 Animals and test chemicals

Juvenile common carp (*C. carpio*) of approximately 6 months old, with mean weight of 2.5 ± 0.48 g (mean ± SEM, *n* = 360) and mean fork length of 49 ± 4.3 mm (mean ± SEM) were held in aerated tanks in a flow-through water system, at 22 °C, maintained under a 16:8 light:dark cycle and fed daily *ad libitum* with pelleted feed (Biomar Incio Plus 0.8 mm). Fish were supplied by the husbandry unit at AstraZeneca, Brixham and were acclimated for 8 weeks prior to being transferred into experimental tanks at the start of the exposure experiment.

Test compounds were all obtained from Sigma–Aldrich, Poole, UK, unless stated otherwise.

### 2.2 Experimental design

Carp were exposed to CFA (CAS: 882-09-7) via a flow through system. On day zero, carp were randomly allocated to one of three duplicated treatment groups; 20 mg/L CFA, 4 μg/L CFA or dilution water controls; in 9 L glass tanks at a density of 10 fish per tank. There were 3 sampling time points, giving a total of 6 tanks per treatment. Two separate stock solutions were prepared for the two dosing regimens. For the high (pharmacological) concentration exposure (20 mg/L), a 2.5 g CFA/L stock solution was prepared in reverse osmosis (RO) water using 0.6 g/L NaOH in order to assist dissolution. For the 4 μg/L concentration exposure, a 2.5 mg CFA/L stock was prepared from the first solution. These stock solutions were diluted in dechlorinated tap water and delivered to each mixing cell at appropriate flow rates to give the desired nominal concentrations of 20 mg/L and 4 μg/L, respectively. The CFA solutions were delivered to the individual tanks at a rate of 60 mL/min. To balance the concentration of NaOH across all treatments, a stock of RO water containing NaOH [0.6 g/L] was also delivered to the dilution water control (containing only dechlorinated tap water) and 4 μg/L treatments. The stock solutions were replaced every two days. Water was maintained at 22 ± 0.2 °C (mean ± SEM), with water temperature, dissolved oxygen and pH measured in all tanks at least twice weekly during the exposure period (Table 2).

### 2.3 Sampling

Duplicate tanks of fish were sampled at day 4 and day 10 of the exposure. The remaining fish were then depurated for a further 4 days (exposed to dilution water only) and sampled on day 14. On each sampling day, 20 fish (10 from each replicate tank) were sampled from each treatment group. Fish were anaesthetized in MS222 solution (500 mg/L in dechlorinated water, adjusted with 1 g/L NaHCO_3_ to pH 7.3), weighed, fork length measured and blood collected from the caudal vein using heparinized (5000 U/mL) capillary tubes. Blood was centrifuged immediately (7000 × *g*, 4 min; Haematokrit 210, Hettich) and plasma separated, snap frozen and stored at −80 °C until further analysis. Fish were then humanely killed according to UK Home Office regulations, and the liver dissected out, weighed, snap frozen and stored at −80 °C until required.

### 2.4 Water and plasma analysis

CFA was measured in water samples from each individual tank, and in the plasma isolated from each sampled fish, using liquid chromatography with tandem mass spectrometry (LC–MS/MS). Water and plasma samples were prepared using acetonitrile and an internal standard added prior to analysis.

#### 2.4.1 Sample preparation: water samples

For dilution water control and low dose (4 μg CFA/L) samples, 800 μL of each water sample was transferred to a 96 deep well plate. 200 μL of acetonitrile, containing 50 nM of internal standard was added prior to analysis by LC–MS/MS. For high dose (20 mg CFA/L) samples 50 μL of each water sample was transferred to a vial containing 10 mL of 10 nM internal standard in 80:20 water:acetonitrile. 1 mL was then transferred to a 96 deep well plate for analysis by LC–MS/MS.

#### 2.4.2 Sample preparation: plasma samples

10 μL plasma from individual fish in the high dose (20 mg CFA/L) samples, or 50 μL plasma (pooled from five fish) for dilution water control and low dose (4 μg CFA/L) samples were added to a 96 deep well plate along with 490 μL acetonitrile and the sample extracted using a Genogrinder (Spex) at 1000 stokes/min for 3 min. The plate was centrifuged at 4000 rpm for 30 min and 200 μL of supernatant removed and evaporated to dryness (Turbovap). The residue was re-suspended in 80:20 water: acetonitrile; 200 μL for the low dose and dilution water control samples and 400 μL for the high dose samples (equivalent to ×10 or ×100 dilution, respectively); containing 10 nM internal standard, ready for analysis by LC–MS/MS.

#### 2.4.3 Instrumental analysis

LC–MS/MS analyzes were performed using a CTC PAL autosampler (Thermo) with a MS gradient pump (Thermo) interfaced to a TSQ Quantum Access triple quadrupole mass spectrometer (Thermo) equipped with a heated ESI probe. Chromatographic separation was achieved using a Hypersil Gold C18 column (50 × 2.1 mm, 3 μM (Thermo) running gradient elution at 500 μL/min. Eluents were: (A) water and (B) methanol, both containing 0.1% of formic acid. Gradient elution was applied as follows: For water samples, 0 min 20% B; 1.5 min 100% B; 3 min 100% B and 3.01 min 20% B and for plasma samples, 0 min 20% B; 1.5 min 20% B; 10 min 100% B; 12.5 min 100% and 12.51 min 20% B. For water samples, retention time of CFA and the internal standard was 1.9 min and 1.6 min, respectively, while for plasma samples they were 6.4 min and 5.5 min. The mass spectrometer was operated in negative ion, electrospray ionization mode using the following parameters: Capillary temperature – 270 °C; vaporizer temperature – 350 °C; spray voltage – 2850 V; sheath gas – nitrogen at 50 (arbitrary units) and auxilary gas – nitrogen at 30 (arbitrary units).

Compound detection was by selected reaction monitoring (SRM) using argon at 1.5 torre as a collision gas and the following transitions monitored; For CFA, the precursor ion was the deprotonated molecular ion *m*/*z* = 213 and the product ion was *m*/*z* = 127 at a collision energy of 18 V. For the internal standard, the precursor ion was the deprotonated molecular ion *m*/*z* = 406 and the product ion was *m*/*z* = 172 at a collision energy of 31 V. Quantitation was by peak area with reference to standards of known concentration of CFA using an internal standard method.

### 2.5 mRNA analyzes

#### 2.5.1 RNA extraction and reverse transcription

Frozen 10 mg aliquots of liver were homogenized directly in Tri-reagent (Chomczynski, 1993) and total RNA was isolated following manufacturer’s instructions. The amount of RNA was quantified using a NanoDrop spectrophotometer and RNA quality was determined both by electrophoresis on an ethidium bromide-stained 1.5% agarose gel and through the measurement of A260/A280 ratio. 1 μg RQ1 DNase treated (Promega) total RNA was subsequently reverse transcribed to cDNA using random hexamers (Eurofins MWG Operon) and MMLV reverse transcriptase (Promega), according to the protocol described previously (Filby and Tyler, 2005).

#### 2.5.2 Real-time quantitative polymerase chain reaction (RT-qPCR)

RT-qPCR was carried out on cDNA samples for each of the treatments at each of the three sampling points (*N* = 8 for each treatment) for the target genes using Absolute QPCR SYBR Green Fluorescein mix (ABgene) according to the protocol described previously (Filby and Tyler, 2005). Briefly, primer pairs were optimized for annealing temperature (Ta), specificity confirmed by melt curve analysis, and the detection range, linearity and amplification efficiency (*E*) established using serial dilutions of carp liver cDNA. RT-qPCR was carried out using Absolute QPCR SYBR Green Fluorescein mix (ABgene), with an initial activation step of 95 °C for 15 min followed by 30–40 cycles of denaturation (95 °C, 10 s) and annealing (appropriate Ta, 45 s) and final melt curve analysis. Ribosomal protein 8 (*rpl8)* was used as a ‘housekeeping’ gene, to normalize the target gene expression, using a development of efficiency correlated relative quantification as described previously (Filby and Tyler, 2005), as it was found not to alter following exposure to CFA (*p > 0.05*) (Corcoran et al., 2012). Details of primers used for RT-qPCR are shown in Table 1.

### 2.6 Biochemical analyzes

#### 2.6.1 Acyl coA-oxidase (Acox1) activity

Acox1 (EC 1.3.3.6) activity was quantified in liver homogenates for each sample (*N *= 8 per treatment per sampling point) by fluorometrically measuring the production of the reactive oxygen species (ROS) hydrogen peroxide (H_2_O_2_; generated specifically by the peroxisomal β-oxidation pathway) using a modification of the method described previously (Poosch and Yamazaki, 1986). Lauroyl-CoA was used as the enzymatic substrate and production of H_2_O_2_ via the direct transfer of electrons to oxygen by Acox1 determined by measurement of the oxidation of 4-hydroxyphenylacetic acid to a fluorescent product (6,6′-dihydroxy-(1,1′-biphenyl)-3,3′;-diacetic acid) in a horseradish peroxidase-coupled reaction. 10 mg frozen liver aliquots were homogenized in buffer containing 0.3 M mannitol, 10 mM HEPES, 1 mM EGTA (pH 7.2) in a hand-held homogenizer. Homogenates were centrifuged at 3000 × *g* for 5 min to remove unbroken tissue and heavy mitochondria. Assays were performed in a 96-well plate with 10 μL of each sample added to wells containing 50 μL of assay cocktail comprised of 60 mM potassium phosphate buffer (pH 7.4) containing 4 U/mL horseradish peroxidase, 1 mM 4-hydroxyphenylacetic acid, 100 μM lauoryl-CoA, 20 μM flavin adinine dinucleotide (FAD) and 0.2 mg/mL triton X-100. Samples were added to wells with assay cocktail containing no substrate as controls. Samples were added under indirect light as FAD is light sensitive, incubated in the dark at 37 °C for 30 min and the reactions terminated by the addition of 1.5 mL of chilled 100 mM sodium carbonate buffer (pH 10.5) containing 2 mM potassium cyanide. All samples were analyzed in triplicate. Fluorescence was measured at room temperature with excitation at 318 nm and emission at 405 nm (Tecan MS200); the difference in fluorescence with and without lauroyl-coA in assay cocktail was used to indicate Acox1 activity. H_2_O_2_ concentrations were determined relative to a standard curve of known amounts of H_2_O_2_ incubated with substrate free assay cocktail. H_2_O_2_ standards were made daily by serial dilution of 30% H_2_O_2_ in deionized water. Protein concentrations in the cell homogenates were determined for each sample using Bradford reagent (an assay based on Bradford, 1976) and bovine serum albumin (Fisher) as reference standard protein. Acox1 activity was expressed as activity/min/mg protein.

#### 2.6.2 Cu,Zn superoxide dismutase (SOD1) activity

Sod1 (EC 1.15.1.1) activity was measured in liver cytosol for each sample (*N *= 8 per treatment per sampling point) according to the protocol described previously (Porte-Visa et al., 2005) based on inhibition of the reduction of nitro-blue tetrazolium (NBT) by superoxide, using xanthine as the substrate. Frozen samples of liver (∼10 mg) were homogenized in 1 mL PBS (pH 7.4), centrifuged at 14,000 × *g* for 10 min and the supernatant (cytosol) separated to measure SOD1 activity only; SOD1 makes up 90% of cellular SOD and is present in the cytosol whereas SOD2 is present in the mitochondrial fraction. The cytosol fraction was diluted 1:10 in PBS and 25 μL loaded into a 1 mL cuvette with 1.25 μL xanthine oxidase and 965 μL substrate solution containing 0.1 mM xanthine, 0.1 mM EDTA, 0.05 mg BSA/mL and 0.025 mM NBT. The absorbance at a wavelength of 560 nm was then recorded spectrophotometrically and units of Sod1 activity calculated by comparison to a standard curve of Sod1 activity using known concentrations of Sod1 between 0.001 and 1 U/mL. Protein concentrations were determined for each sample using Bradford reagent (as above) and SOD1 activity was expressed as units of enzyme activity/min/mg protein.

### 2.7 Data analysis

Data are presented throughout as mean ± standard error of the mean (SEM). All statistical analyzes were carried out using SigmaPlot^®^ software (Systat Software, Inc., Chicago, USA). Data were tested for normality/equality of variances, and log transformed if necessary. Effects of test compounds on levels of gene expression, enzyme activity, and morphological endpoints were determined using one-way ANOVA followed by Fisher LSD multi comparison procedure, where appropriate. This study was not intended as a toxicological assessment of CFA, but rather was essentially comprised of two separate experimental paradigms focused on a pharmacological or environmentally-relevant exposure level, and thus treatment responses were compared directly with the relevant control groups.

Where data did not meet the assumptions of normality and/or homogeneity of variance, data were analyzed using Kruskal–Wallis one-way ANOVA on ranks followed by Dunn’s *post hoc* analysis. In all cases, *p *< 0.05 was considered statistically significant.

## 3 Results

### 3.1 CFA concentrations in the exposure water and fish plasma

During the exposure, mean measured water concentrations of CFA were 4.61 ± 0.58 μg/L and 18.58 ± 4.5 mg/L (mean ± SE, *n* = 18) for the 4 μg/L and 20 mg/L nominal tanks, respectively (Table 2). After 4 days of depuration, CFA concentrations in the water were below the limit of quantification (1.3 μg/L) in all tanks.

Concentrations of CFA measured in the plasma of exposed carp are summarized in Table 3. In the lower (environmental) exposure treatment (4 μg/L) and dilution water control groups plasma levels were below the limit of quantification (20 μg/L) across all time points measured. In the pharmacological concentration exposure group (20 mg/L) after 4 days exposure, the blood plasma levels of CFA were 5.54 ± 0.45 mg/L (approximately 28% of the measured water levels) and after 10 days exposure there was little, if any change in this concentration (6.11 ± 0.43 mg/L; approximately 30%). After 4 days of depuration, plasma levels were below the limit of quantification (20 μg/L) in all treatment groups. The mean recovery from fortified plasma samples was 103%, with a relative standard deviation (RSD) of 3.0.

### 3.2 Morphometric parameters

There was no significant effect of CFA concentration on the size, growth or condition of the fish at any of the time points sampled (Table 3). There was also no effect of the treatment on hepato-somatic index (HSI). Blood hematocrit was significantly elevated in plasma from fish exposed to the pharmacological CFA concentration at day 4 (ANOVA, *p *< 0.05) but there were no significant effects at any of the other time points or treatment levels.

### 3.3 Hepatic transcript levels of PPARα-related genes

**Fig. 1:**
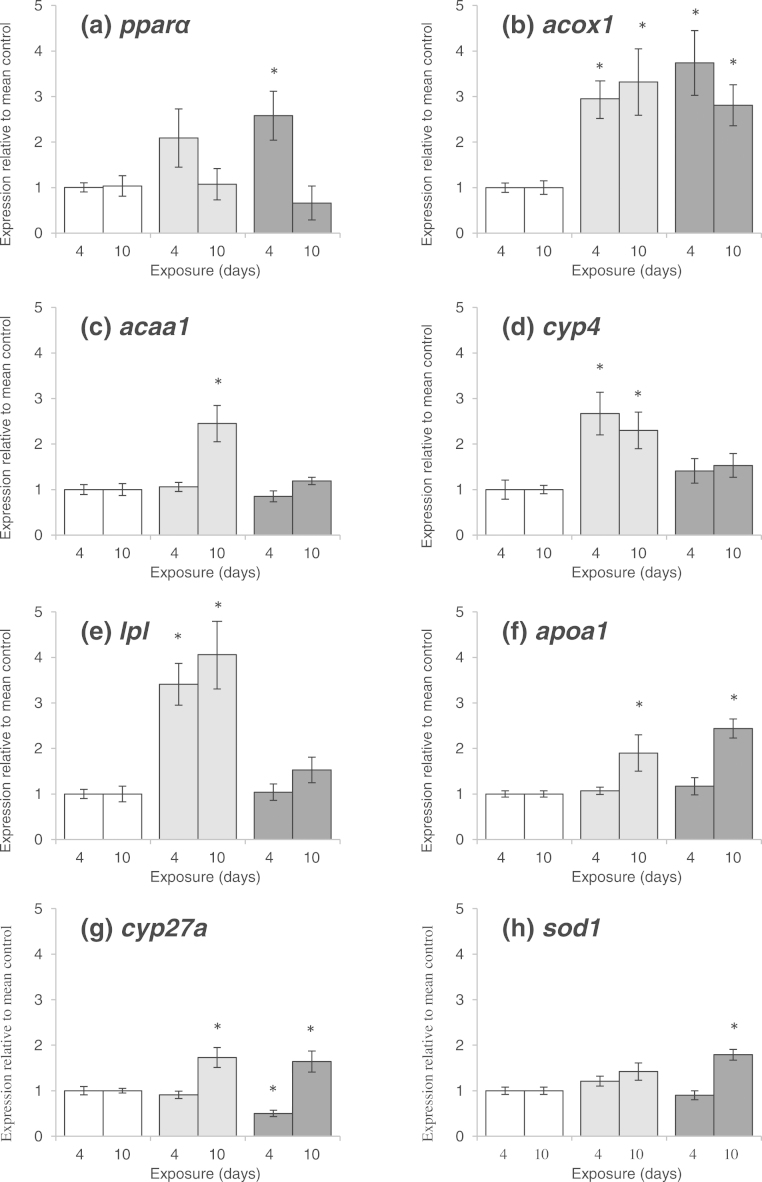
mRNA levels of genes associated with PPARα activation in carp exposed *via* the water to clofibric acid at 20 mg/L (dark grey bars) and 4 μg/L (light gray bars) and to a dilution water control (white bars). Data are presented as mean fold difference relative to the mean control at the respective time point. Fish were sampled at day 4 and day 10 of the exposure. *N* = 8 for each treatment. Error bars represent SEM. An asterisk above the bar indicates a significant difference compared to the control group (*p *< 0.05).

Fig. 1 shows transcript levels of a suite of genes known to be regulated by PPARα in mammals, measured in carp exposed to the two concentrations of CFA for 4 or 10 days. Levels were also measured after 4 days depuration; however there was no significant difference in mRNA levels for any of the genes measured across the treatments at this time point (*i.e.*, no treatment effects; data not shown).

After 4 days of exposure, *pparα* transcript levels were significantly higher in the fish exposed to 20 mg/L compared with the control animals (approximately 2.5-times control levels). *Acox1* transcript levels were significantly higher at both exposure concentrations, with mRNA levels reaching almost 4 times higher than the control levels in the 20 mg/L treated fish. Transcript levels of *cyp4* and *lpl* were significantly higher in the 4 μg/L exposed animals only (2.5-times and 3.5-times, respectively), and in contrast *cyp27a* mRNA levels were significantly reduced, by approximately twofold in the pharmacological exposure group relative to controls levels. There was no significant change in transcript levels of *acaa1, apoa1* or *sod1* after 4 days of exposure*.*

After 10 days of exposure *acox1, apoa1* and *cyp27a* transcript levels were significantly higher relative to control at both exposure concentrations (up to 3.5, 2 and 2-times, respectively). *Acaa1, cyp4* and *lpl* mRNA levels were increased relative to controls at 4 μg/L only (2.5, 2.5 and 4-fold, respectively) and *sod1* transcription was higher (by 2-times control) at 20 mg/L only. There was no change in the transcript levels of *ppara* for either exposure level.

### 3.4 Acyl-coA oxidase (Acox1) and Cu,Zn superoxide dismutase (Sod1) activity

**Fig. 2:**
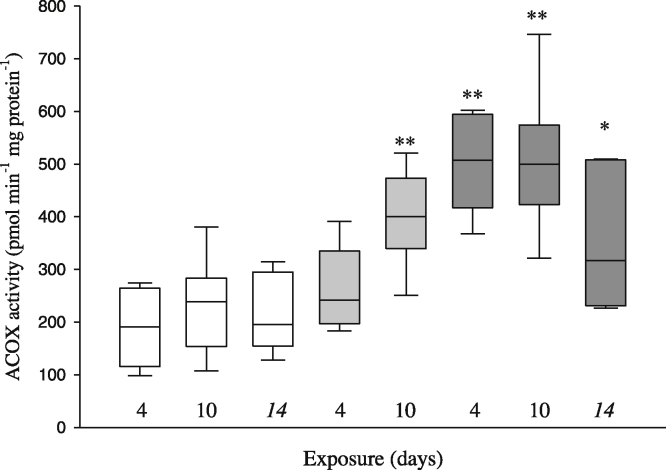
Activity of acyl-coA oxidase (expressed in pmol peroxisomal H_2_O_2_ production per minute per mg protein) in carp exposed *via* the water to clofibric acid at 20 mg/L (dark grey boxes) and 4 μg/L (light grey boxes) and to a dilution water control (white boxes) after 4 and 10 days exposure and a further 4 days depuration (day 14). There were no depuration data for 4 μg/L treatment. Activity is expressed as pmol H_2_O_2_ per minute per mg protein as described in methods. *N* = 8 in each case. Box represents inter-quartile range, bars represent maximum and minimum values and horizontal line represents median value for each treatment. Asterisks above boxes indicate significant differences compared with the control group (*p < 0.05, **p < 0.001).

After 4 days of exposure to CFA, Acox1 activity was significantly higher (2.5-times higher) in the 20 mg/L treated animals compared with controls and this was also the case in both treatment groups after 10 days (between approximately 2- and 2.5- fold higher; Fig. 2). Interestingly, even after 4 days of depuration, Acox1 activity remained significantly higher (approximately 2-times) in the 20 mg/L treated animals relative to controls.

**Fig. 3:**
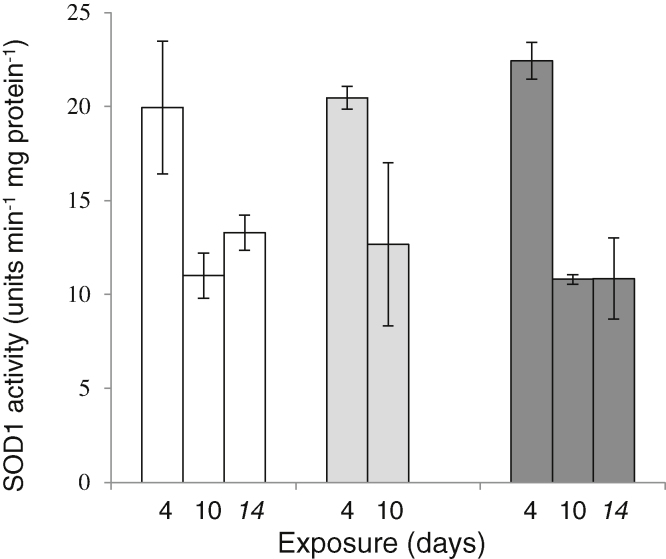
Enzyme activity of Cu,Zn superoxide dismutase (SOD1) in livers of carp exposed to CFA [20 mg/L (dark grey bars), 4 μg/L (light gray bars) and dilution water controls (white bars) after 4 and 10 days exposure and after 4 days depuration (day 14). SOD1 was measured *via* inhibition of the reduction of NBT and expressed as units of SOD1 activity per minute per mg protein. One unit of SOD activity was defined as the amount of enzyme resulting in 50% inhibition of NBT reduction as calculated by use of a standard curve with known amounts of SOD. Error bars represent standard error. *N* = 3 for each treatment. There was no low concentration exposure group for day 14. There were no significant differences between time points.

There was no significant effect of CFA treatment on Sod1 activity at any sampling point during the exposure or depuration (Fig. 3).

### 3.5 Transcript levels of genes with products involved in hepatic biotransformation

**Fig. 4:**
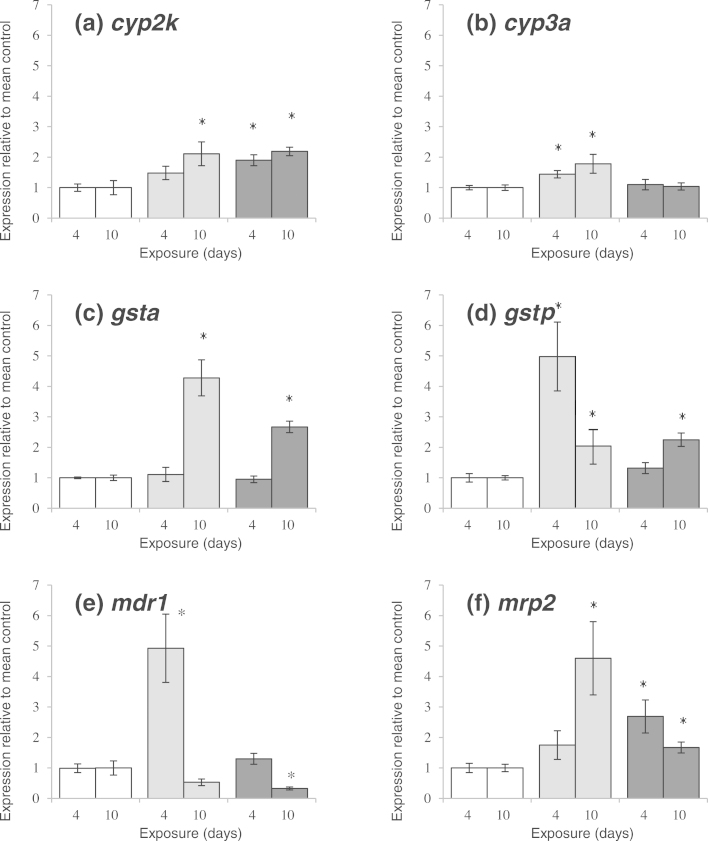
mRNA levels of genes involved in xenobiotic metabolism in carp exposed *via* the water to clofibric acid at 20 mg/L (dark grey bars) and 4 μg/L (light grey bars) and to a dilution water control (white bars). Data are presented as mean fold difference relative to the mean control at the respective time point. Fish were sampled at day 4 and day 10 of the exposure. *N* = 8 for each treatment. Error bars represent SEM. An asterisk above the bar indicates a significant difference compared to the control group (*p *< 0.05).

After 4 days of exposure to CFA, all of the analyzed drug biotransformation and transport genes (*cyp2k*, *cyp3a*, *gstp*, *mdr1, mrp2*), except for *gsta*, showed significantly higher transcript levels compared with controls at one or both treatment levels (Fig. 4). The patterns shown differed between genes, however: *cyp2k* and *mrp2* mRNA levels were significantly higher at 20 mg/L (by 1.9 and 2.7 times, respectively); whereas *cyp3a, gstp* and *mdr1* mRNA levels were elevated only at 4 μg/L (1.4, 5 and 5-times, respectively). There was no effect on the transcription of *gsta* after 4 days of exposure.

After 10 days of exposure to CFA, transcript levels of all hepatic biotransformation genes were significantly elevated at one or both treatment levels compared with the respective control, with the exception of *mdr1.* Conversely, *mdr1* mRNA levels were significantly lower (3 times) than the controls at 20 mg/L CFA.

Most gene transcripts showed significantly higher levels at both 4 μg/L and 20 mg/L with the exception of *cyp3a* which was elevated only at 4 μg/L (1.8 times relative to control). The greatest changes observed were for *gsta* and *mrp2* mRNA where in the 4 μg/L exposure group transcription was 4.3 and 4.6 times those of control levels, respectively.

No significant difference in transcript levels occurred across any of the genes measured after 4 days of depuration (data not shown).

## 4 Discussion

In mammals, PPARα activation results in modified transcript levels of various genes associated with the inducible β-oxidation pathway in peroxisomes (and to a lesser extent in mitochondria). These include the key genes acyl-coA oxidase (*acox1*), enoyl-coenzyme A hydratase/3-hydroxyacyl coenzyme A dehydrogenase (*ehhadh)*, and thiolase (*acaa1*), as well as fatty acid transport protein (*fatp1*) and long-chain acyl-coA synthetase (*acs*). In mammals, upregulation of these genes collectively serves to increase fatty acid uptake, conversion and oxidation in the liver, resulting in a lower availability of fatty acids for triglyceride synthesis (Staels et al., 1998).

In fish, less is known of the function of PPARs (Ibabe et al., 2004), although both PPARα and PPARβ have been shown to exhibit activation profiles to a range of ligands known to activate the mammalian counterparts (Cocci et al., 2013), and a comparable role in lipid regulation is likely from the data generated thus far (Prindiville et al., 2011; Venkatachalam et al., 2012). Here we exposed carp to two concentrations of the PPARα ligand CFA: 20 mg/L and 4 μg/L which were, respectively, selected to investigate the responsiveness of carp to PPARα activation at a pharmacologically-relevant concentration, and to assess whether such activation could occur at CFA concentrations similar to those previously measured in the environment.

Acox1 induction, in particular, is a key, rate-limiting step in the peroxisomal β-oxidation pathway in mammals, and is also suggested to be a rapid and specific marker of exposure of aquatic organisms to PPs (Cajaraville et al., 2003). We found transcript levels of *acox1*, and to a lesser extent *acaa1*, were increased on exposure of carp to CFA supporting activation of the PPARα pathway, at the concentration of 4 μg CFA/L comparable with levels measured in effluents discharged into the environment. Furthermore, elevated levels of *acox1* mRNA coincided with increased Acox1 enzyme activity, in agreement with previous studies on other fish species, including increased *acox1* mRNA levels in zebrafish (Venkatachalam et al., 2012) and elevated Acox1 enzyme activity in both fathead minnow (Weston et al., 2009) and salmon hepatocytes (Ruyter et al., 1997) after exposure to CFA.

In humans, several members of the cytochrome P450 4 (CYP4*)* family are known to be transcriptionally regulated by PPARα. These enzymes have an important role in microsomal ω-oxidation of fatty acids (Hardwick, 2008) and there is a close association between induction of CYP4 and activation of the peroxisomal fatty acid metabolizing system (Simpson, 1997). In fish, a link between CYP4 and PPARα has not been established. In the present study, the effects of CFA on *cyp4* mRNA levels are not easily explained as there was an apparent increase in expression upon CFA exposure at the lower exposure concentration, but not at the higher exposure. In a previous study in *Fundulus heteroclitus*, exposure to 10 μg CFA/L did not result in alteration of CYP4A protein levels after 17 days of exposure (Emblidge and DeLorenzo, 2006).

In line with the induction of hepatic peroxisomal β-oxidation in rodents (but not in humans) it is well documented that PPs, including fibrates, increase peroxisomal number and volume (peroxisome proliferation) which can in turn result in oxidative stress and in some cases hepatocarcinogenesis (Rao and Reddy, 1991). In rainbow trout hepatocytes *in vitro*, it has been shown that CFA and the related fibrate ciprofibrate, but not gemfibrozil, increase the activity of peroxisomal Acox1 and that there is a strong correlation between induction of Acox1 activity and the relative amount of peroxisomal bifunctional enzyme after exposure to any of these three compounds (Donohue et al., 1993). Similarly, *in vivo* exposures of rainbow trout to the lipid regulating drugs ciprofibrate or gemfibrozil resulted in an increase in Acox1 enzyme activity and induction of oxidative stress (Yang et al., 1990), or an increase in liver/body weight ratio (Scarano et al., 1994), respectively. Although peroxisomal parameters were not measured directly here, the absence of changes in HSI here suggests increased liver volume in carp is not significant following exposure to CFA. The resultant oxidative stress associated with peroxisome proliferation is thought to be due, in part, to the increased peroxisomal production of H_2_O_2_ via increased Acox1 activity (Varanasi et al., 1996). Consequently, we also measured *sod1* transcript and Sod1 activity as markers of oxidative stress (Fridovich, 1975), particularly as *sod1* has been identified as a gene target of PPARα and responds to fibrate treatment in rodents (Yoo et al., 1999; Wang et al., 2010). We observed a modest increase in *sod1* mRNA levels on day 10 at the pharmacological exposure concentration, which was not, however, reflected by elevated enzyme activity, perhaps suggesting alternative antioxidant pathways were activated, such as catalase, glutathione peroxidase or glutathione-S-transferase (see later) (Oruc et al., 2004).

The level of *ppara* transcription is believed to be auto-regulated in mammals (Torra et al., 2002). Here *ppara* gene transcript levels were significantly increased after 4 days of exposure to CFA, but not after 10 days, despite a number of other downstream target gene transcripts remaining elevated. It may be the case that there was a transient activation of transcription during the period of the exposure study, with a peak in expression after 4 days and a subsequent return to basal expression by day 10. This hypothesis aligns with a previous study in fathead minnow where it was found that *ppara* gene expression was not significantly elevated after 21 days of exposure to 108.9 mg/L CFA, despite an increase in activity of Acox1 (Weston et al., 2009).

Therapeutically, in addition to reducing triglyceride synthesis, fibrates have been shown to alter lipoprotein metabolism and cholesterol homeostasis in humans, via a number of PPARα regulated targets, including the apolipoproteins AI and AII (APOA1 and APOA2), lipoprotein lipase (LPL) (Staels et al., 1998), as well as various enzymes involved in bile acid synthesis (cholesterol catabolism) and transport in the liver (Kersten, 2008; Li and Chiang, 2009). Interestingly, however, the *Apoa1* gene in rodents is unresponsive to PPARα activation (Vu-Dac et al., 1998). Here both *lpl* and *apoa1* mRNA levels were up-regulated in carp exposed to CFA suggesting that the transcription of both genes is activated by PPARα, as is the case in humans. This response pathway in fish has not been fully investigated, however teleost apolipoproteins including *apoa1* are known to be regulated by exposure to oestradiol-17β and are thought to play a role in vitellogenesis (Levi et al., 2009). The elevation of *lpl* mRNA levels at the environmental CFA treatment concentration only is also curious. In a previous study, exposure of rainbow trout to the related fibrate gemfibrozil resulted in elevated levels of the *lpl* gene transcript, but not Lpl enzyme activity and there was no evidence of the activation of PPARs despite decreased plasma lipoprotein levels (Prindiville et al., 2011). The pattern exhibited here with *lpl* (as well as *cyp4* and *acaa1* mentioned above) mRNA, is the opposite to that of *ppara* mRNA levels, perhaps reflecting indirect downstream activation of lipoprotein/cholesterol regulation, negative feedback on *ppara* transcription, or merely a reflection of the time-lag between receptor and downstream pathway activation. More work is required to fully understand the response shown here.

In mammals, it has been demonstrated that fibrates disrupt bile acid synthesis via the PPARα mediated suppression of cholesterol 7α-hydroxylase (*Cyp7a1*) and sterol 27-hydroxylase (*Cyp27a*) (Post et al., 2001). CYP7A1 catalyzes the first and rate-limiting step in the classical pathway, converting cholesterol into 7α hydroxyl cholesterol; CYP27A mediates sterol side chain oxidation in the classic pathway and catalyzes the first two steps of the alternative pathway. The simultaneous inhibition of these enzymes after long term fibrate treatment has been linked to imbalanced bile acid and cholesterol secretions in humans (Pullinger et al., 2002). In our study, *cyp27a* mRNA levels were reduced in carp after 4 days of exposure to 20 mg/L CFA, which is what would be expected based on human function. However, after 10 days of exposure, *cyp27a* transcript levels were significantly increased at both exposure concentrations; the opposite of what might be expected. Interestingly, the pattern shown by the *cyp27a* transcript mirrors *apoa1* transcript levels, which would be expected to increase (Li and Chiang, 2009), and could support an adaptive increase in bile acid synthesis as a result of elevated cholesterol concentrations.

After 4 days of exposure to CFA, the overall *in vivo* hepatic mRNA level profiles in carp for the drug biotransformation and transporter genes *cyp2k*, *cyp3a*, *gsta, gstp,* *mdr1* (*abcb1)* and *mrp2* (*abcc2*), were generally in agreement with data we previously generated in an *in vitro* carp hepatocyte preparation (Corcoran et al., 2012). All of the gene transcripts measured showed a significant increase in levels at one or both treatment concentration with the exception of *gsta*. Similarly, after 10 days transcription of all genes was elevated at one or both treatment concentrations, with the exception of *mdr1* which was significantly down-regulated. This again broadly mirrors data previously generated in our *in vitro* carp hepatocyte preparation after exposure to CFA (Corcoran et al., 2012) and further supports a role for PPARα in the regulation of a number of xenobiotic metabolizing enzymes in fish, as is hypothesized in mammals.

As previously mentioned, Sod1 enzyme activity was unaltered in our CFA-exposed animals. It may be that alternative oxidative stress pathways were activated by exposure to CFA and the measured increased Acox1 activity. For example here *gsta* and *gstp* transcript levels were elevated in a number of CFA-treated groups compared with controls. In European eel (*Anguilla anguilla*) exposed to the fibrate gemfibrozil, there was little change in Gst activity, but there was an increase in catalase activity (Lyssimachou et al., 2014), another important mediator of cellular oxidative stress regulation. Taken together, this could suggest that activation of the teleost PPARα results in hepatic oxidative stress necessitating the activation of at least some cellular antioxidant defenses.

Collectively our data demonstrate that exposure of common carp to the PPARα ligand CFA affects the level of transcripts of a number of genes known to be regulated by PPARα in mammals, and results in altered activity of the key peroxisomal enzyme Acox1, a biomarker of exposure to PPs.

Determining a causal relationship between instantaneous measures of gene expression (at the time of termination) and the resultant protein product is problematic, and the complexity of the kinetics of synthesis and degradation adds to this difficulty (Vogel and Marcotte, 2012). The paradigm used here, however, was to examine selected nodal genes (Yu et al., 2007) believed to be directly involved with the PPARα signaling pathway. Detecting changes in the levels of transcript of these genes in relation to those shown by control animals would strongly suggest that the PPAR axis is under the influence of clofibrate in the fish. However, although these findings support a role for PPARα in lipid metabolism in fish, as is the case in mammals, a direct link in terms of PPARα activation and altered biological function have yet to be demonstrated.

Measured plasma concentrations in the pharmacological exposure group were 6 mg/L (approximately 30% of the measured water concentration) which is at least 10 times lower than the therapeutic plasma concentrations of CFA reported in humans (Regenthal et al., 1999). As such the observed hepatic gene and enzyme responses suggest that the carp PPARα and associated pathways appear relatively sensitive to activation by CFA. Furthermore, several of these transcriptional responses occur at a water concentration of 4 μg/L, comparable with some of the concentrations reported in effluents discharged into the aquatic environment and a little below the 28 day growth NOEC of 10 μg/L reported for trout (Owen et al., 2010). The plasma concentrations measured are in line with those previously measured in rainbow trout plasma (Owen et al., 2010) suggesting a similar level of bioavailability, however, the degree and type of plasma protein binding remain unknown in fish. The relatively similar plasma concentrations of CFA at the 4 and 10 day measurements indicate a low propensity for bioconcentration in this tissue, unlike for gemfibrozil in goldfish (Mimeault et al., 2005). Moreover, effective depuration of CFA from the plasma, as illustrated after transfer of exposed fish into clean water for 4 days, would suggest a relatively short half-life for CFA in plasma in carp.

As previously highlighted, CFA is only one of a number of fibrate drugs to have been detected in the environment, for example fenofibrate, bezofibrate and ciprofibrate have all been measured at similar concentrations in surface waters (Runnalls et al., 2007). This raises the possibility of additive effects due to strong similarities in their mechanisms of action. Moreover, many other compounds classed as PPs and acting via similar mechanisms are present in the aquatic environment including herbicides, plasticizers and wood pulp compounds, adding to the possible mixture effects of PPs on these target endpoints in fish.

As a final note, the study presented here suggests that PPARα may be involved in regulating the transcription of a number of important biotransformation genes in carp, in common with what is known in mammals. This potentially extends the role of the PPARα to not only a regulator of lipid homeostasis, but also a key modulator of hepatic xenobiotic metabolism.

## Figures and Tables

**Table 1:** Target genes and details of primers and assays used with RT-qPCR analysis. Ta is annealing temperature; PCR efficiency represents the ‘*E*’ value.

Gene		Accession number	Primer direction	Primer sequence (5′–3′)	Ta (°C)	*E*	Product size (bp)
Peroxisome proliferator-activated receptor α	*ppara*	FJ849065	Sense	GGGAAAGAGCAGCACGAG	62.0	2.032	105
			Antisense	GCGTGCTTTGGCTTTGTT			
Acyl coA-oxidase	*acox1*	CF660510	Sense	ACAGCACAGCAAGAGCAATG	59.0	1.971	104
			Antisense	ACAGAGTGGACAGCCGTATC			
Thiolase	*acaa1*	See^a^	Sense	TTGCCTGTGGTGTGGAG	59.0	2.200	90
			Antisense	CAACATTCTCTGAGGTTATTCC			
Cytochrome P450 4	*cyp4*	GU046698	Sense	TTGACCTCTGCCACTTG	57.0	2.110	138
			Antisense	CTGATAACTTCCGCTGTATG			
Lipoprotein lipase	*lpl*	FJ716101	Sense	TTGGGTTACAGTCTTGGTGCTC	62.0	2.110	104
			Antisense	AAAGGGCATCATCGGGAGAAAG			
Apolipoprotein A1	*apoa1*	AJ308993	Sense	GCCGAAGAAGGTGAAGC	57.0	2.012	82
			Antisense	GGTGGCAAGGAAGAAAGG			
Sterol 27α hydroxylase	*cyp27a*	CF660988	Sense	GAGCCACGAAAGTTCAAACC	56.0	2.012	88
			Antisense	CATCTCCAGTTCAGCAATGC			
Cu,Zn superoxide dismutase	*sod1*	CA964628	Sense	GGAATACTCGGTCATTGG	54.0	2.036	100
			Antisense	ACTGAGTGATGCCTATAAC			
Cytochrome P450 2 K	*cyp2k*	GU046696	Sense	GCTCTTCCTGTTCTTC	60.0	2.070	103
			Antisense	TGTGACTTCTACTACTC			
Cytochrome P450 3 A	*cyp3a*	GU046697	Sense	CCAAGGACCACAAGAAGAAG	60.0	1.921	159
			Antisense	AGCCGCCGAAGATGAAG			
Glutathione S-transferase α	*gsta*	DQ411310	Sense	TACAATACTTTCACGCTTTCCC	61.5	1.979	149
			Antisense	GGCTCAACACCTCCTTCAC			
Glutathione S-transferase π	*gstp*	DQ411313	Sense	GTCCTTTGCTCTGCCTCTCTG	60.5	2.103	141
			Antisense	TTACTGCTTGCCATTGCCATTG			
Multidrug resistance 1 (ATP-binding cassette sub-family B member 1)	*mdr1*(*abcb1*)	AY999964	Sense	TTGCGGCTGTGGGAAGAG	58.5	2.104	109
			Antisense	GTGGATGTTCAGTTGCTTTGTG			
MDR related protein 2	*mrp2*(*abcb1*)	AY679169	Sense	TTCGGCTCTAATCTGGATG	58.5	2.080	149
			Antisense	CTCACCCGCTGTTTCTG			
Ribosomal protein 8	*rpl8*	See (Bickley et al., 2009)	Sense	CTCCGTCTTCAAAGCCCATGT	60.0	2.140	N/A
			Antisense	TCCTTCACGATCCCCTTGATG			

a CarpBase, http://legr.liv.ac.uk/carpbase/index.htm (Williams et al., 2008).

**Table 2:** Nominal and measured water concentrations of CFA and pH and oxygen saturation during the 10 day exposure period and subsequent 4 day depuration. Measured concentrations, oxygen saturation and pH values are given as mean ± standard error. There was no depuration period for the 4 μg/L treatment group.

	Nominal water concentration of CFA		
	Control	4 μg/L	20 mg/L
Measured concn. (mean ± SE, *n* = 18)	<LOQ^a^	4.61 ± 0.58	18.58 ± 4.51
% Nominal concn.	–	115.25	92.90
Measured concn. during depuration	<LOQ	–	<LOQ
pH (mean ± SE, *n *= 18)	7.61 ± 0.16	7.51 ± 0.04	7.63 ± 0.01
O_2_% sat. (mean ± SE, *n* = 18)	79.07 ± 0.86	80.54 ± 0.72	79.95 ± 0.75

Measured concentration values are presented in μg/L for the 4 μg/L group and mg/L for the 20 mg/L group. ^a^LOQ = limit of quantification LOQ = 1.3 μg/L.

**Table 3:** Fish morphometric and physiology data. All data presented as mean ± standard error; HSI = hepatic somatic index, calculated as liver weight/fish wet weight; condition factor (*K*) calculated as (fish wet weight/(fork length^3^)); Haematocrit calculated after centrifugation of the blood in capillary tubes (see Section 2) and defined as a ratio of red blood cell (RBC) volume to total blood volume, and expressed as a percentage of total blood volume. LOQ = limit of quantification (20 μg/L).

	Nominal water concentration CFA							
	Control			4 μg L^−1^		20 mg L^−1^		
	Day 4	Day 10	Day 14	Day 4	Day 10	Day 4	Day 10	Day 14
Plasma [CFA] (μg/L)	<LOQ	<LOQ	<LOQ	<LOQ	<LOQ	5537 ± 450	6113 ± 430	<LOQ
Liver weight (g)	0.043 ± 0.01	0.052 ± 0.02	0.053 ± 0.01	0.052 ± 0.01	0.065 ± 0.02	0.048 ± 0.01	0.049 ± 0.01	0.061 ± 0.01
Fish wet weight (g)	2.40 ± 0.77	2.83 ± 0.67	3.07 ± 0.57	3.14 ± 0.82	3.78 ± 0.82	2.48 ± 0.47	2.924 ± 0.64	3.46 ± 0.54
HSI	1.87 ± 0.29	1.85 ± 0.35	1.83 ± 0.36	1.71 ± 0.33	1.74 ± 0.54	1.98 ± 0.42	1.70 ± 0.46	1.77 ± 0.33
Fork length (mm)	48.55 ± 5.32	51.44 ± 3.76	52.99 ± 3.61	53.61 ± 4.92	55.82 ± 3.64	49.24 ± 3.81	52.04 ± 3.87	55.19 ± 3.55
Condition factor (K)	2.04 ± 0.13	2.04 ± 0.13	2.05 ± 0.05	2.01 ± 0.16	2.15 ± 0.13	2.07 ± 0.02	2.05 ± 0.16	2.05 ± 0.14
Haematocrit % (RBC:total blood)	33.49 ± 1.48	35.69 ± 0.74	34.74 ± 1.19	34.53 ± 1.01	35.74 ± 0.89	37.69 ± 0.90*	36.62 ± 0.89	33.00 ± 1.04

* Indicates significant difference to control value (*p *< 0.05).
